# Caffeine for Headaches: Helpful or Harmful? A Brief Review of the Literature

**DOI:** 10.3390/nu15143170

**Published:** 2023-07-17

**Authors:** Anna Zduńska, Joanna Cegielska, Sebastian Zduński, Izabela Domitrz

**Affiliations:** 1 Department of Neurology, Faculty of Medicine and Dentistry, Medical University of Warsaw, 01-809 Warsaw, Poland; anna.zdunska@wum.edu.pl (A.Z.); izabela.domitrz@wum.edu.pl (I.D.); 2 Medical Rehabilitation Facility, The National Institute of Medicine of the Ministry of Interior and Administration, 02-507 Warsaw, Poland; sebastian.zdunski@cskmswia.gov.pl

**Keywords:** caffeine, migraine, caffeine-withdrawal headache, tension-type headache, hypnic headache, post-dural puncture headache, spontaneous intracranial hypotension, medication overuse headache

## Abstract

Consumption of caffeine in the diet, both daily and occasional, has a significant biological effect on the nervous system. Caffeine, through various and not yet fully investigated mechanisms, affects headaches. This is especially noticeable in migraine. In other headaches such as hypnic headache, post-dural puncture headache and spontaneous intracranial hypotension, caffeine is an important therapeutic agent. In turn, abrupt discontinuation of chronically used caffeine can cause caffeine-withdrawal headache. Caffeine can both relieve and trigger headaches.

## 1. Introduction

Caffeine, a naturally occurring methylxanthine, is probably the most commonly consumed psychoactive compound worldwide. Caffeine is found primarily in coffee, but also in tea, chocolate and energy drinks. Consuming moderate amounts of caffeine, i.e., 200–400 mg of caffeine per day (about 4–5 cups of coffee), is safe for healthy, non-pregnant adults. Caffeine consumed once in a dose of 50–100 mg increases energy, alertness, reaction accuracy and the ability to concentrate and focus attention, and it improves well-being and mood and reduces fatigue. It also improves physical fitness, short-term memory and cognitive performance. However, high doses of caffeine (400–800 mg once) can have negative effects and evoke anxiety, nervousness, tremors, insomnia or tachycardia [1]. Therefore, caffeine, especially in excess, is not recommended for people with hypertension or a history of seizures and should not be recommended for them [2]. Even therapeutic doses of caffeine have been associated with atrial fibrillation and central nervous system toxicity and should be used with caution in high-risk patients. These statements do not apply to caffeine used occasionally, in small amounts [3].

Caffeine content varies depending on the type of food you consume. For example, filtered coffee contains 85 mg of caffeine per 125 mL of drink, instant coffee contains 65 mL of caffeine in the same volume, tea contains 32 mg of caffeine per 150 mL of drink, energy drinks contain 80 mg/330 mL, dark chocolate contains 60 mg of caffeine per 30 g and milk chocolate contains only 6 mg per 30 g [1]. 

After oral administration, caffeine is absorbed quickly and completely; its bioavailability is almost 100%. In addition, it freely penetrates the blood–brain and placental barriers, and its half-life varies between 4 and 9 h [4].

Caffeine, whose structure is similar to that of adenosine, affects pain control. Adenosine is an inhibitor of neuronal activity in the central (CNS) and peripheral nervous system (PNS). Adenosine causes different effects in the central and peripheral nervous system, related to its effect on adenosine receptors. Three subtypes of these receptors are known: A1, A2 (A2A and A2B) and A3 receptors. They are expressed in various parts of the human nervous system, both central and peripheral. Thus, for example, activation of A1 and A2A adenosine receptors in neuropathic pain results in antinociceptive activity. Due to its similar structure to adenosine, caffeine may compete with adenosine for A2A receptors, causing their inhibition [5]. Caffeine may also develop an analgesic effect due to its ability to inhibit the synthesis of leukotrienes and prostaglandins [4]. These two mechanisms, i.e., receptor antagonism and a multi-site effect on eicosanoid pathways (mainly by modulating cyclooxygenase activity), may explain the antinociceptive and adjuvant effects of caffeine [5].

As experimental data showed, caffeine administered in doses from 25 to 100 mg/kg has an intrinsic antinociceptive effect [6]. It has been found that adding small doses of caffeine to a standard dose of common painkillers reinforces their analgesic effect [7]. Low doses of caffeine are used as a fortifying adjuvant in combination with antidepressants, paracetamol, acetaminophen and non-steroidal anti-inflammatory drugs. Many complex analgesics with such a combination of active substances are available on the market, also without a prescription [5]. It is known that higher concentrations of discussed in this article methylxanthine, unattainable with a standard diet, may cause further biological/biochemical effects, i.e., the release of Ca^2+^, inhibition of phosphodiesterase or blocking GABA A receptors, and thus additionally affect the perception of pain [8].

An important function of adenosine in the CNS is its participation in the regulation of sleep and wake processes. Adenosine exhibits sleep-promoting activity by inhibiting the release of excitatory neurotransmitters, which results in a reduction in cortical excitability. Caffeine, by inhibiting the adenosine A2A receptors, induces a state of wakefulness [6]. Even low concentrations of this natural metyloksntine, achieved after drinking a single cup of coffee, result in significant antagonism of A1 and A2A adenosine receptors and may result in increased alertness. Caffeine contributes to an increase in cortical excitability, increases alertness and improves cognition.

Adenosine also has a vasodilating effect in the brain by binding to the A2A and A2B receptors located in the smooth muscle of the cerebral vessels, probably mainly by opening ATP-dependent K+ channels and reducing Ca^2+^ conductivity. The involvement of other mechanisms is possible [9]. Caffeine has the opposite effect—it narrows blood vessels [4].

There is growing evidence that not only environmental but also genetic factors contribute to habitual caffeine consumption. Knowledge of genetic determinants can inform about the causal role of coffee and other beverages in health and identify people most susceptible to the health consequences of regular consumption [10]. Genetic factors affecting the variability of caffeine metabolism were identified and the modulatory role of systemic caffeine levels in caffeine consumption behavior was demonstrated. This large individual variability in caffeine metabolism may modify the potential adverse or beneficial effects of coffee on health [11]. Recent genome-wide association studies (GWASs) from populations of European descent identified single-nucleotide polymorphisms (SNPs) in AHR (aryl-hydrocarbon receptor) and CYP1A1/1A2 (cytochrome P450 1A1 and 1A2) genes that are associated with habitual consumption of caffeine and coffee. CYP1A2 is the major caffeine-metabolizing enzyme and AHR is an upstream inducer of CYP1A1 and CYP1A2 transcription [12]. Cornelis et al. conducted a genome-wide (GW) meta-analysis, mainly with regard to regular coffee consumption (cups per day); confirmed loci were examined for putative functional and biological significance. Eight loci met the significance with an effect size per allele of 0.03–0.14 cups per day. Six loci are located near genes potentially involved in pharmacokinetics (ABCG2-ATP Binding Cassette Subfamily G Member 2, AHR-Aryl Hydrocarbon Receptor, POR-P450 Oxidoreductase, CYP1A2-Cytochrome P450 Family 1 Subfamily A Member 2) or pharmacodynamics (BDNF-Brain-Derived Neurotrophic Factor, SLC6A4-Solute Carrier Family 6 Member 4) of caffeine. Two mapped genes, GCKR (Glucokinase Regulator) and MLXIPL/CHREBP (MLX Interacting Protein-Like/Carbohydrate-Response Element-Binding Protein), are associated with caffeine metabolic features, but their roles in caffeine consumption are unknown. SNP alleles nearby GCKR, MLXIPL, BDNF and CYP1A2 were associated with higher coffee consumption [13]. Future research on SNPs in AHR and CYP1A1-CYP1A2 should investigate whether these gene variants actually affect the caffeine metabolic rate [12]. In addition, twin studies have shown that the heritability of caffeine-related traits ranges from 0.36 to 0.58 [14].

The paper reviews publications on caffeine and headaches. The current information on the possible mechanisms of caffeine’s effect on migraine and other headaches is summarized and the data on caffeine as a therapeutic agent in these diseases are reviewed. The availability of caffeine in the diet as a headache trigger, as well as the possibility of its therapeutic or prophylactic use in some headaches, make it a substance worthy of greater scientific attention.

## 2. Material and Methodology

This literature review includes all articles on the association between caffeine and different types of headaches. Clinical databases including PubMed, MEDLINE and Google Scholar were searched from 1990 to May 2023. The following combinations of keywords were used to search the potential literature: ‘caffeine/coffee’ and ‘headache’ or ‘migraine’ or ‘tension type headache’ or ‘hypnic headache’ or ‘post-dural puncture headache’ or ‘postoperative headache’ or ‘spontaneous intracranial hypotension’. Other keywords were also used: ‘caffeine-withdrawal headache’, ‘medication overuse headache’, ‘chronic daily headache’, ‘migraine trigger factors’ and ‘migraine treatment’. 

The inclusion criteria were as follows: (1) only English-language articles; (2) all types of articles: clinical trials, observational, cross-sectional, case–control study and case reports studies; (3) human-based research only; (4) literature based on the adult population only. 

Some studies were excluded due to the following: (1) failure to include caffeine in the factors provoking migraine attacks (only general dietary factors); (2) failure to include caffeine as a treatment modality; (3) individual studies/articles originally published before 1990; (4) duplicate articles, reviews or conference papers; (5) articles on, among others, the pediatric population.

## 3. The Role of Caffeine as a Factor Involved in the Pathophysiology of Headaches, Triggering Headache Attacks and an Agent Used in Their Treatment

### 3.1. Migraine

According to the International Classification of Headache Disorders, 3rd ed., migraine is recurrent headache disorder manifesting in attacks lasting 4–72 h. A typical migraine headache is unilateral, throbbing and moderate to severe in intensity. It is aggravated by routine physical activity and is accompanied by nausea and/or photophobia and phonophobia. Chronic migraine is defined as a headache that occurs on 15 or more days per month for more than 3 months; on at least 8 days a month, the pain must have the typical features of a migraine headache [15].

Long-term consumption of caffeine in migraine patients triggers a cascade of physiological processes that can result in three different clinical situations: worsening of the original headache, headaches associated with caffeine withdrawal (e.g., weekend migraine attacks) and headaches caused by overuse of painkillers containing caffeine. Caffeine can both relieve and trigger migraine attacks. Habitual caffeine consumption is associated with migraine and the development of chronic daily headaches [4]. 

Peripheral antinociception, achieved by activation of the A1 receptor located on peripheral sensory nerve endings and in lamina II of the spinal cord, is suggested to be mediated by blocking the release of substance P and endogenous calcitonin gene-related peptide (CGRP). Experimental data indicate that there are also A2A receptors on peripheral nerve endings, the blocking of which can also cause antinociception. The blocking of this receptor by caffeine consumed in ordinary doses may possibly be partly responsible for caffeine’s analgesic effects in humans. Central dopaminergic mechanisms may also be involved in this effect, as caffeine increases the release of dopamine [6].

The prodromal or warning phase of a migraine is usually defined as a period of 2–48 h before an aura or migraine headache with symptoms indicating an impending headache attack. This phase is reported by 30% to 90% of migraine patients [16], and the most commonly described symptoms of them are neck stiffness, dizziness, fatigue, changes in mood and sensitivity to light or sound, yawning and drowsiness [17]. The intensity of as many as 30.8% to 57.2% of these symptoms was defined as moderate or severe [16]. These symptoms are similar to caffeine withdrawal syndrome, which is characterized by drowsiness, difficulty concentrating, mood swings and nausea. It is not uncommon for these clinical problems to be accompanied by muscle pain or stiffness. The observations cited above suggest that the same or similar pathophysiological pathways may be involved in both the prodromal phase of migraine and caffeine withdrawal syndrome. However, sensory hypersensitivity, the main feature of migraine, is absent in caffeine withdrawal syndrome. The overlapping of symptoms may be related to the fact that caffeine withdrawal is a factor that triggers a migraine attack [6]. The higher frequency of migraines reported on weekend mornings may, at least in part, be related to the effects of caffeine withdrawal [18].

However, the mechanisms underlying the abnormal excitability of the cerebral cortex and the cyclical nature of migraine attacks are still insufficiently understood. The pathophysiological role of the hypothalamus is increasingly taken into account, especially since functional imaging studies indicate its increased activity both in the prodromal phase of migraine and during a headache attack [6]. There is also a clear link between sleep disturbances and migraine attacks. Abnormal sleep patterns predispose one to develop migraines. Although the risk of a migraine attack is low during sleep, it increases in the morning, especially after a sleepless night or poor night’s sleep. Brennan et al., while studying families with a sleep disorder called familial advanced sleep phase syndrome, found that mutations in the gene casein kinase Iδ that cause sleep problems also seem to cause migraines. In two families, all members with a sleep-disrupting mutation in the casein kinase Iδ gene suffered concurrent migraines [19].

Especially when used continuously, caffeine’s stimulating effect and its prolonging of the state of wakefulness may contribute to sleep disorders, and consequently to triggering migraine attacks also in this mechanism. Due to the structural similarity of caffeine and adenosine mentioned earlier, the results of studies on the effect of adenosine on the development of migraine seem to be significant. There are reports in the literature of elevated blood adenosine concentrations during migraine attacks, and headaches are frequently reported after intravenous administration of adenosine during cardiological treatment. Intravenous adenosine has also been reported to trigger migraine attacks [6]. A systematic review of preclinical studies on the role of adenosine in the pathophysiology of migraine by Thuraiaiyah et al. shows that adenosine receptors modulate pain transmission in the trigeminal vascular system. The adenosine A1 receptor has an inhibitory effect, while the stimulation of the A2A receptor causes vasodilation and activation of the trigeminal nerve pain pathway. Adenosine causes vasodilation and modulates CGRP release. Based on the results of the above and other publications, it seems that the antagonization of adenosine by caffeine may have a positive effect on the development of migraine attacks, and chronic caffeine consumption in humans increases the migraine burden [20].

It is generally accepted that gastric stasis or some form of delayed gastric emptying occurs during an acute migraine attack. In a study by Aurora et al., gastric motility was assessed using gastric scintigraphy in patients with migraine. It has been shown that in patients with migraine, gastric stasis occurs both during the acute migraine attack and in the interictal period. At the same time, it has been suggested that the nausea is caused by a central process and not by gastric stasis itself [21]. The mere reduction in gastric motility during a migraine attack slows down the absorption of painkillers and, consequently, reduces their effectiveness. Caffeine, in turn, stimulates the work of the stomach, which in the case of migraine patients taking analgesics with the addition of caffeine, may have significant clinical implications—it may increase the effectiveness of these preparations and improve their analgesic effect also in this mechanism [22]. Probiotic supplementation has been shown to modulate migraine attacks; probiotics can also increase the rate of gastric emptying and relieve gastric stasis [23]. Caffeine can directly affect gastrointestinal physiology by increasing intestinal motility and reducing intestinal transit time. González et al. confirmed that regular coffee consumption can affect the composition of the intestinal microflora, and caffeine intake was directly related to fecal Bacteroides levels [24].

Consumption of caffeine in higher doses causes an acute diuretic effect and, consequently, may lead to dehydration [25]. The diuretic effect of caffeine is due to its antagonism to adenosine. Adenosine reduces natriuresis and diuresis, mainly through competitive inhibition of the A1 receptor in the cells of the renal proximal tubules [26]. Dehydration is one possible migraine trigger. Despite the known link between dehydration, hypohydration and headache, the pathophysiology is still not fully understood. The mechanism underlying dehydration headache is likely to be variable and multifactorial [27]. A cross-sectional study conducted by Khorsha and al. examined the association between water intake and migraine in 256 women aged 18–50 years with migraine. Women who drank more water and total fluids had statistically significantly lower severity of migraine disability, pain severity, headache frequency and duration of headaches [28]. At the same time, dehydration may occur secondary to an acute migraine attack or a migraine status due to nausea, impaired food intake and vomiting. However, in a randomized controlled study of 50 migraine patients in the emergency department, which assessed the headache response of 1 L of saline over 1 h versus saline at 10 mL/h for 1 h, no significant difference was found in headache severity between groups. Although this study did not demonstrate a therapeutic role for intravenous fluids in the treatment of severe migraine attacks, this issue should be further investigated taking into account the degree of dehydration of patients [29].

In addition to its diuretic and dehydrating effects, caffeine increases urinary excretion of potassium, sodium, calcium and magnesium [26]. In the context of migraine, urinary magnesium loss is probably of the greatest importance. Research conducted in recent years shows that hypomagnesemia may be of significant importance in initiating migraine attacks, especially migraine with aura [30]. Changes observed in migraine (especially migraine with aura), such as regional disturbances in cerebral blood flow and spreading cortical depression, as well as oxidative stress with brainstem activity, underlying the pathogenesis of migraine, may be related to electrolyte disturbances and the aforementioned magnesium deficiency [31].

Triggers for a headache attack are those that, alone or in combination, trigger headache attacks in susceptible individuals and usually precede the attack by less than 48 h [32]. Retrospective surveys and cross-sectional studies are the most commonly used methods to identify headache triggers [17]. Caffeine is rarely reported as a migraine trigger in these studies [33]. Coffee as a migraine trigger is indicated in the literature with a frequency of 6.3% to 25.4% [34].

In a study by Andress-Rothrock et al. on a group of 200 migraine patients, 8% of patients reported caffeine as a trigger for a headache attack [35]. In a Korean study conducted on a group of 62 patients with episodic migraine who kept a headache diary in an app for 3 months, caffeine was identified as a provoking factor by 2.4% of patients. Headaches with triggers were associated with greater pain intensity, headache-related disability and medication failure than those without triggers [36]. In a study in Asia of 684 headache patients (319 migraine patients and 365 tension-type headache patients), dietary triggers were identified in 37.3% of patients, with 44.2% of migraine patients and 31.2% of TTH patients having dietary triggers. The most common dietary trigger for headache was coffee (19.9%), which was also significantly associated with migraine (25.4%) compared to TTH (15.1%) [37]. A cross-sectional study of headache triggers was conducted by Wöber et al. at a headache center with 120 subjects, including 66 migraine patients and 22 tension-type headache patients. Caffeine was a headache trigger in about 10% of patients [38]. In a study conducted by Chądzyński et al. in Poland, in a group of 40 women with episodic migraine, 4.5% of patients indicated caffeine as a factor provoking a migraine attack [39].

When considering the effects of caffeine on migraine, two therapeutic aspects should also be mentioned. On the one hand, it is known that frequent use of caffeine can cause addiction and trigger withdrawal headaches. On the other hand, the addition of caffeine increases the effectiveness of painkillers in acute migraine attacks. Thus, in a prospective study by Lee et al., caffeine withdrawal improved the response to pharmacological treatment of migraine with triptans [40]. The study was conducted in a group of migraine patients who consumed caffeine-containing beverages daily. They were instructed to stop taking caffeine. It turned out that withdrawal from caffeine was independently associated with a very good effect of rescue treatment: as many as 72.2% of people in the abstinence group reported success with triptan treatment, compared to only 40.3% of people in the non-abstinence group. At the same time, the initial dose of caffeine had no effect on the withdrawal effect. This suggests that both high-caffeine and low-to-moderate caffeine users may benefit from caffeine withdrawal. The results indicate that caffeine withdrawal (when taken chronically) may be beneficial in the treatment of migraine attacks, regardless of the degree of baseline caffeine saturation. It is believed that the long-term use of caffeine may lead to the upregulation of adenosine receptors and a compensatory increase in plasma adenosine, a potent vasodilator. Excess adenosine can have the effect of accelerating migraine headaches. Therefore, daily caffeine consumption may reduce the effectiveness of triptans (which are associated with vasoconstriction). While caffeine withdrawal can be beneficial in treating migraines, it can be complicated by another type of headache: caffeine withdrawal pain. This pain occurs in the mechanism of “rebound” cerebral vasodilation and subsides after 2 weeks of abstinence from caffeine [41].

Caffeine-containing pain medications are used frequently by headache patients. In migraine patients, caffeine is used primarily as an adjuvant, i.e., an agent that enhances the effects of standard painkillers. In studies, caffeine combinations of paracetamol, acetaminophen, acetylsalicylic acid and ibuprofen showed significantly better effectiveness in the emergency pharmacotherapy of tension headaches and migraine attacks compared to their decaffeinated counterparts, with favorable tolerance in the vast majority of patients. The most common adverse reactions were nervousness (6.5%), nausea (4.3%), abdominal pain/discomfort (4.1%) and dizziness (3.2%). A dose of caffeine of 130 mg increases the effectiveness of analgesics in TTH, and doses ≥ 100 mg increase the benefit in migraine [42]. 

The good efficacy of a combination of aspirin (500 mg), acetaminophen (500 mg) and caffeine (130 mg) in the treatment of migraine attacks was demonstrated by Lipton et al. in three double-blind, placebo-controlled, parallel-group studies involving 1220 migraine patients. Headache reduction or resolution was observed in 59% of patients taking caffeine-combination painkillers, and in 33% of patients in the placebo group [43]. In a multicenter, double-blind, randomized, parallel-group, placebo-controlled study, a combination product containing 250 mg acetaminophen, 250 mg aspirin and 65 mg caffeine or 200 mg ibuprofen or equivalent placebo was used. A total of 1555 migraine sufferers were included in the analysis. The caffeine-containing combination was shown to provide significantly higher efficacy and faster headache relief compared to ibuprofen [44]. Goldstein compared a combination of migraine medications (acetaminophen 500 mg, aspirin 500 mg, and caffeine 130 mg) with sumatriptan 50 mg in aborting a migraine attack. The combination of acetaminophen, aspirin and caffeine was found to be more effective than sumatriptan in the early treatment of migraine [45]. An Italian multicenter, randomized, double-blind study showed good efficacy and tolerability of a combination of paracetamol 1000 mg and caffeine 130 mg compared to sumatriptan 50 mg in the treatment of migraine attacks. Both drugs reduced headaches to a similar extent and were well tolerated by patients [46]. 

In a multicenter, double-blind, crossover clinical trial conducted on 229 patients in Spain, patients were treated with two attacks of moderate to severe migraine: almotriptan 12.5 mg or ergotamine 2 mg in combination with caffeine 200 mg. Almotriptan was found to be a more effective and safer drug in aborting a migraine attack [47]. A randomized, double-blind, crossover study evaluated the efficacy of 100 mg diclofenac sodium capsules with or without 100 mg caffeine versus placebo in patients during migraine attacks. Diclofenac in combination with caffeine was effective in treating a migraine attack in 41% of patients, diclofenac alone in 27% of patients and placebo in only 14% of patients. Diclofenac in combination with caffeine turned out to be the most effective [48]. 

In a study by Baratloo et al., the efficacy of intravenous caffeine citrate 60 mg versus magnesium sulfate 2 g was evaluated in the treatment of acute migraine headache in 70 patients total, with 35 patients in each group. Both intravenous caffeine citrate and intravenous magnesium sulfate significantly reduced the severity of migraine pain, but the magnesium sulfate group showed greater improvement [49]. Studies evaluating the role of caffeine as an analgesic in the acute treatment of migraine are summarized in Table 1.

### 3.2. Tension Type Headache (TTH)

According to the International Classification of Headache Disorders, 3rd ed., a tension-type headache is a recurrent headache disorder with episodes lasting from 30 min to 7 days. The typical characteristics of this headache are bilateral location, pressing or tensing (non-throbbing) nature, mild to moderate intensity and no correlation of severity with routine physical activity. It is not accompanied by nausea or vomiting, but photophobia or phonophobia may occur (but never both at the same time) [15].

A multicenter, double-blind, placebo-controlled parallel study by Diamond et al. on a group of 301 patients demonstrated the efficacy and safety of ibuprofen in combination with caffeine in the treatment of tension-type headache. Comparisons were made between ibuprofen alone, caffeine alone, ibuprofen plus caffeine and placebo. According to previous data, caffeine showed analgesic properties, although the effects were not as significant as the combination of ibuprofen and caffeine [50]. In turn, in a study conducted in Italy on a group of 93 patients with tension-type headache, a good safety profile and tolerability of the combination of paracetamol 1000 mg and caffeine 130 mg were demonstrated, compared to naproxen 550 mg and placebo. Paracetamol with caffeine was well tolerated and effective in the treatment of acute TTH [51]. In a meta-analysis by Diener et al. of studies on the use of a combination of acetylsalicylic acid (500 mg), acetaminophen (500 mg) and caffeine (130 mg) in the treatment of an acute episode of TTH, this combination of drugs was shown to be well tolerated and effective in 28.5% of patients, while the effectiveness of acetaminophen was revealed in 21% of patients, and in the case of placebo, only 18% of people in this group improved [52].

## 4. The Role of Caffeine as the Main Component in the Treatment of Headaches

### 4.1. Hypnic Headache (HH)

According to the International Classification of Headache Disorders, 3rd edition, a hypnic headache is a recurrent headache that develops only during sleep and causes awakening. The headache lasts from 15 min to 4 h after waking up. A diagnosis of hypnic headache can be made if the characteristic headache occurs at least 10 days a month for more than 3 months [15]. This type of primary headache usually starts after the age of 50. Its prevalence among patients treated in headache centers is estimated at 0.07–0.35%, but the exact prevalence of hypnic headache is unknown [53].

One of the typical features of HH is that caffeine often stops acute pain, but also when used prophylactically before going to bed, it prevents the onset of pain [8]. Some authors consider a good response to caffeine as a pathognomonic feature of HH [54]. It should be added that experimental studies have shown that the administration of adenosine A2A receptor agonists into the subarachnoid space may induce NREM sleep [55].

Due to the rarity of hypnic headache, treatment recommendations are based mainly on expert opinions and small case series [56]. Based on the review of the available literature, it seems that caffeine can be used both as a first-line drug in the treatment of the acute phase of HH and as a prophylactic agent. Almost all patients reported that drinking a cup of strong black coffee for pain was effective in treating acute HH attacks at night. In contrast, drinking a cup of coffee before going to bed effectively prevented headache attacks. Caffeine-containing painkillers also seem to be effective in the treatment of hypnic headache—patients typically report that the use of a caffeinated combination of painkillers (e.g., paracetamol with caffeine) leads to significant pain relief during an acute HH attack, while painkillers alone do not stop the headache [8].

In a retrospective study by Tariq et al., 40 patients treated for HH in a reference headache center were described. Of the 15 different drugs tried, the best response was seen with lithium, with 70% complete response and 20% moderate response. In the case of caffeine, a complete response was obtained in 28% of patients and a moderate response in 43% of patients [57].

With regard to hypnic headache, it can be said that the therapeutic response to caffeine goes beyond the usual analgesic effect seen in other types of headache [8].

### 4.2. Post-Dural Puncture Headache (PDPH)

Post-dural puncture headache may be a consequence of a lumbar puncture performed for diagnostic or therapeutic purposes, or may occur as a complication of epidural anesthesia. It rarely occurs in an intense form, but can be nagging [58]. According to the International Classification of Headache Disorders, 3rd ed., PDPH is included in the group of headaches attributed to low cerebrospinal fluid pressure. Headache develops within 5 days of the dural puncture [15].

The pathogenesis of PDPH remains unclear, but it is assumed that it is probably a consequence of leakage of CSF into the epidural space and reduction of CSF pressure. There are two possible mechanisms. First, lowering the pressure of the cerebrospinal fluid causes traction of pain-sensitive intracranial structures in the vertical position, which causes the characteristic headache. Second, the loss of cerebrospinal fluid causes compensatory venous dilatation (Monro–Kelli principle) responsible for headache [59]. The occurrence of post-puncture headache is influenced by the needle size, direction of bevel, needle design, replacement of the stylet and number of lumbar puncture attempts [3]. A characteristic feature of PDPH is a reduced scale of pain in the lying position and its increase after verticalization [60]. Methylxanthines, such as caffeine and theophylline, are some of the best-studied medications for PDPH relief. These drugs act symptomatically in PDPH by two mechanisms: they interfere with calcium uptake in the sarcoplasmic reticulum and block phosphodiesterase and antagonize adenosine, which causes cerebral vasoconstriction, and they increase the production of cerebrospinal fluid by stimulating sodium-potassium pumps [2]. 

Caffeine is known to have been used to treat PDPH as early as 1949 [61]. Several studies and some case reports recommend oral and intravenous caffeine as a treatment option, although headache recurrence after caffeine treatment is common [3]. In a randomized, double-blind clinical trial by Yucel et al., 1000 mL of saline and 500 mg sodium caffeine benzoate or 1000 mL of saline were administered within the first 90 min after spinal anesthesia. Caffeine administration reduced the incidence of PDPH [62]. However, Stevens et al. noted that the study did not take into account all the criteria for the diagnosis of post-puncture headache and previous caffeine consumption by the subjects, which is why some patients may have developed caffeine-withdrawal headache rather than post-puncture headache [63]. In a study by Camann et al., 300 mg of caffeine orally was administered to 40 postpartum PDPH patients and pain scores were assessed immediately before drug administration, and after 4 and 24 h. Oral caffeine relieved the headache [64]. Esmaoglu et al., in a randomized, double-blind, placebo-controlled study of 210 otherwise healthy patients scheduled for spinal anesthesia for elective lower limb surgery, failed to demonstrate the efficacy of a multi-dose combination of caffeine and paracetamol. In the study, patients received a placebo, 500 mg paracetamol + 75 mg caffeine or 500 mg paracetamol + 125 mg caffeine. Perhaps the doses of caffeine used were too low [65]. 

Studies in patients with PDPH have shown that the use of a single dose of 300 mg of oral caffeine or 500 mg of intravenous caffeine relieves headache associated with this syndrome. A Cochrane review concluded that these doses of caffeine were better than placebo in the treatment of PDPH, with fewer patients requiring additional interventions [42].

The administration of caffeine appears to be a non-invasive and safe option that can replace the use of invasive methods, e.g., epidural blood patch [61]. The currently recommended dose for the treatment of post-puncture headache is 300–500 mg of oral or intravenous caffeine once or twice daily. However, therapeutic doses may be associated with toxicity to the central nervous system (pathological agitation, anxiety) or the heart (atrial fibrillation), which should be taken into account [59].

### 4.3. Spontaneous Intracranial Hypotension (SIH)

Spontaneous intracranial hypotension is an important secondary headache syndrome that has been increasingly recognized in recent years. Headache usually starts when standing and goes away when lying down (referred to as orthostatic headache) [66]. According to the International Classification of Headache Disorders, 3rd ed., headache attributed to SIH is included in the group of headaches attributed to low cerebrospinal fluid (CSF) pressure. Headache occurs despite the absence of a procedure or trauma known to be able to cause CSF leakage. It develops in temporal relation with the occurrence of CSF low pressure or with CSF leak, or leads to its detection [15].

It often has a sudden onset and is accompanied by other neurological symptoms: dizziness, tinnitus, mental stupor, and behavioral changes, but clinical variability makes diagnosis difficult [67]. Although positional (usually tilting) headache is the most common symptom of SIH, the positional factor may not be dominant, have variable intensity or evolve. It can also be completely elusive [66]. Data on the exact epidemiology of idiopathic intracranial hypotension are not available, but the annual incidence is estimated at 4–5 cases per 100,000 population. Women aged 35 to 55 are most often affected [67]. 

SIH is caused by a leak of cerebrospinal fluid and is potentially treatable if the leak can be stopped [66]. Loss of cerebrospinal fluid displaces brain structures, causing headache and other neurological symptoms [67]. Most patients have normal CSF pressure, but there is a reduced volume of intracranial CSF. This volume reduction is due to the leakage of cerebrospinal fluid through the meningeal membrane at one or more sites. It is usually caused by one of three causes (etiologies): (1) leaks resulting from the weakening of the dura mater involving nerve root sleeves, (2) tearing of the ventral dura mater associated with disc herniation and (3) fluid (csf)-venous fistulas. Conservative treatment, which includes bed rest, caffeine and hydration, is often recommended as the first-choice therapy for patients with SIH [66].

Caffeine is the most commonly used agent and can be given in the form of caffeinated beverages or caffeine tablets [66]. Firstly, caffeine inhibits phosphodiesterase and antagonizes adenosine, leading to cerebral vasoconstriction; secondly, it increases the production of cerebral fluid by activating the sodium–potassium pump. If symptoms do not respond to medical treatment, an epidural blood patch (EBP) may be considered [68]. In a meta-analysis of studies by D’Anton et al., conservative treatment was effective in 28% of patients. A single epidural blood patch was successful in 64% and large epidural blood patches (>20 mL) had better success rates than small epidural blood patches [69]. Fibrin glue is believed to act as a focal sealer at the site of the leak and is often used in conjunction with EBP, less often as an alternative to EBP. Surgical treatment is considered when the site of the leak has been identified and symptoms persist despite the use of less invasive methods [70].

## 5. The Role of Caffeine Withdrawal in Provoking Headaches

### 5.1. Caffeine-Withdrawal Headache 

The International Headache Society does not list caffeine as a potential cause of analgesic overuse headache, but rather as a substance that can cause headache when regular consumption is abruptly discontinued. Caffeine-withdrawal headache, according to the International Classification of Headache Disorders, 3rd edition, is a headache that develops within 24 h of discontinuation of regular caffeine intake of more than 200 mg/day for at least 2 weeks. It resolves spontaneously within 14 days in the absence of further consumption or within 1 h by intake of caffeine 100 mg [15].

High caffeine intake, especially in coffee, tea, cola and other types of caffeinated beverages, may be a trigger factor for withdrawal headache when caffeine intake is discontinued. The effect of caffeine varies from person to person, for example, due to different body weights or different conditions of absorption, and depends on the developed phenomenon of tolerance. Therefore, it is not possible to establish a uniform, maximum, safe threshold for caffeine consumption [8]. Frequent consumption of caffeine may result in a behavioral disorders called caffeine dependence syndrome, recognized by the WHO as a disease entity. Addiction is caused by increased dopaminergic activity, especially by blocking A2A receptors, resulting in increased dopamine release in the striatum [6]. The long-term effects of caffeine abuse result from the overregulation and hypersensitivity of adenosine receptors. This process may explain the pronounced physical dependence resulting from long-term caffeine abuse. It is also fundamental to understanding withdrawal syndrome: abrupt discontinuation of caffeine intake causes adenosine receptors to become available, leading to vasodilation and a significant increase in blood flow to the brain. These physiological changes are responsible for the caffeine-withdrawal headache [4]. Symptoms such as headache, fatigue and impaired concentration appear between 12 and 24 h after caffeine withdrawal and can occur with chronic caffeine consumption in doses ≥100 mg/day. Withdrawal headaches in caffeine users correspond to increased cerebral blood flow after 20–24 h of caffeine abstinence [9]. 

In an epidemiological study of 1741 people living in the Norwegian town of Vågå, caffeine-withdrawal headache was found in only 7 people (0.4%) with relatively high coffee consumption (average 4.7 cups per day). The coffee relieved that headache. The study was completely retrospective, only looking at spontaneous caffeine intake and withdrawal consequences, including headaches among many symptoms. Conclusions from this study should therefore be drawn with caution [71]. 

In a review of studies on caffeine withdrawal by Juliano et al., the most frequently assessed symptom was headache (48 experimental studies and 6 survey studies). Headache was reported in 77% of the experimental studies that evaluated it. The median percentage of people reporting headache in the experimental studies reviewed was 47%, ranging from 9% to 100% across various ones. In survey studies, the median percentage of caffeine users reporting caffeine-withdrawal headaches was 24%, ranging from 8% to 56%. Abstinence-induced headache has also been shown to be limited in time and resolve rapidly, and often completely, upon re-administration of caffeine [34].

#### Postoperative Headache

Caffeine withdrawal may be one of the factors of the frequently observed perioperative headache, as many operations require patients to fast, which for a significant proportion of patients means coffee-free days [72]. There are no randomized, controlled, multicenter studies evaluating the administration of caffeine in the perioperative period. The results of the few studies conducted suggest that the administration of caffeine in the perioperative period reduces the incidence of headache associated with caffeine withdrawal [73]. Hampl et al. studied 40 patients who underwent minor surgery. Half of the patients were treated with caffeine tablets, and the other half received only placebo tablets. In the placebo group, 50% of the patients reported a headache after surgery, and no one in the caffeine group had a headache [74]. In a prospective, randomized, double-blind clinical trial conducted by Weber et al. in 300 adult patients, the prophylactic postoperative administration of 200 mg intravenous caffeine reduced the incidence of postoperative headache in patients at risk of caffeine withdrawal symptoms [75].

A review of studies by Bright et al. found that withdrawal from caffeine due to hospitalization is rapid; therefore, patients may experience withdrawal symptoms, including headache. Of the seven prospective studies evaluating the effects of caffeine withdrawal, five studies showed that caffeine withdrawal increased the incidence of postoperative headache. Three studies have investigated the administration of caffeine for the relief of postoperative headache, including two prospective randomized controlled trials that concluded that the administration of caffeine prophylaxis reduces the incidence of postoperative headache [76].

## 6. The Role of Caffeine in the Development of Chronic Headache

### 6.1. Medication Overuse Headache (MOH)

Caffeine appears to increase the risk of developing MOH to a level similar to that of ergot derivatives and opioids. Medication overuse headache, according to the International Classification of Headache Disorders, 3rd ed., occurs for ≥15 days/month in a patient with pre-existing headache and is associated with regular overuse for >3 months of one or more medications that may be used for the treatment of primary or secondary/symptomatic headache [15]. It is known that the risk of MOH is related to the frequency of caffeine use [30], but its role in the pathomechanism of MOH remains unclear [8]. 

MOH is characterized by two main pathophysiological mechanisms: cortical hyperexcitability and increased peripheral and central sensitization. Both of these phenomena may be chronic effects of excessive caffeine intake: hyperactivity of the cerebral cortex is induced by increased release of excitatory neurotransmitters (mainly glutamate), while the pronociceptive state is promoted by chronic activation of A2A receptors at the peripheral level. These receptors promote the action of CGRP, a powerful pronociceptive neuropeptide that plays a major role in the transition of headaches to the chronic phase [4]. A retrospective clinical trial conducted at a headache center showed a positive correlation between daily caffeine intake and MOH (increased odds ratio 2.2) as well as chronic migraine (increased odds ratio 2.9) [77].

### 6.2. Chronic Daily Headache (CDH)

A population study by Scher et al. showed a possible relationship between dietary caffeine intake and caffeine use in medicinal products and chronic daily headache. Caffeine consumption for therapeutic and dietary purposes was measured in a group of people suffering from episodic and chronic headaches, including migraine. High therapeutic or dietary caffeine intake at the time of CDH onset was a moderate risk factor for chronic daily headache. Secondary analyses showed that caffeine intake before CDH may be a trigger in a subset of CDH sufferers [78].

## 7. Summary and Conclusions

The links between caffeine and headaches are multidirectional and multifactorial. Caffeine can be both a trigger of a migraine attack, and its withdrawal can provoke a headache (caffeine-withdrawal headache). Appropriately applied doses of caffeine significantly increase the therapeutic effect of common analgesics and NSAIDs in migraine patients and patients with TTH. Conversely, overuse of caffeine-containing medications may expose patients to overuse headache and lead to the development of a chronic form of tension headache or chronic migraine. The important role of caffeine in the treatment of certain types of headaches should also be emphasized: hypnic headache, post-dural puncture headache and spontaneous intracranial hypotension. At the same time, the results of an uncontrolled clinical trial suggest that stopping caffeine consumption may improve the effectiveness of treatment of acute migraine [42]. 

Headache patients may be advised to continue drinking caffeinated beverages as long as they consume low to moderate amounts of caffeine (preferably <200 mg/day) and their daily caffeine intake is similar. If patients cannot consume a similar dose of caffeine throughout the day and maintain a regular level, the best solution is to gradually withdraw from caffeine. Patients should also be advised that the interval between doses of caffeine-containing beverages should not exceed 24 h, as a longer interval may lead to caffeine-withdrawal headache. Patients should also be informed that daily doses of caffeine in excess of 200 mg/day may provoke headaches in some patients [79].

There are other food elements that are used as “medicines” or used complementarily to relieve the ailments and symptoms of various diseases or in any case to treat disorders. The molecular pathways and targets of drugs and nutrients are closely related. Food contains compounds, mostly produced by microorganisms or higher plants, that are capable of producing biological effects that go far beyond nutrition. Many classic drugs are derived from natural compounds [80]. Currently, more and more attention is paid to nutraceuticals, i.e., products that, apart from nutrition, also have medicinal applications. Nutraceuticals can be used to improve health, delay the aging process, prevent chronic disease, increase life expectancy or support the structure and function of the body. Caffeine is considered a nutraceutical, which like other herbal stimulants such as ephedrine, chitosan, mahuang guarana and green tea, are effective in facilitating weight loss in obese people. However, their use is controversial due to potential side effects. Some popular nutraceuticals include ginseng, Echinacea, green tea, glucosamine, folic acid and cod liver oil. Flavonoids are widely distributed in vegetables, onions, chicory, grapefruit, apples, cherries, pomegranates, blueberries and red wine and play a major role in the prevention and treatment of cardiovascular disease. The potential role of nutraceuticals is indicated in the treatment of difficult disorders associated with oxidative stress, including allergies, Alzheimer’s disease, cardiovascular disease, cancer, diabetes, eye diseases, immune disorders, inflammatory diseases and Parkinson’s disease, as well as obesity [81].

Knowledge about the probable, though not fully understood, mechanisms of action of caffeine in headaches and awareness of its role in the treatment of headache patients should be disseminated and contribute to optimal use of this substance. It can help people avoid headaches, limit their triggering and be used to treat certain types of headaches. In clinical practice, certain groups of patients may benefit from the use of caffeine-containing medicines, especially patients with a partial response to simple analgesics and migraine patients with severe nausea associated with gastroparesis. Caffeine is of great importance (as an adjuvant in fixed combinations with analgesics) in the acute management of tension headaches and migraines [42].

The role of caffeine and its safety in medicine require further research. Therefore, caution should be exercised in its use until existing clinical observations are supported by scientific evidence.

## Figures and Tables

**Table 1:** Studies evaluating the role of caffeine as an analgesic in the acute treatment of migraine.

Reference	Method of Study	No. of Patients	Treatments	Outcome
Lipton et al., 1998 [43]	double-blind, randomized, placebo-controlled	1220 (AAC—602, placebo—618)	aspirin 500 mg + acetaminophen 500 mg + caffeine 130 mg (AAC) versus placebo	Patients in the AAC group had a significantly greater reduction in migraine headache compared to those in the placebo group. Pain decreased to mild or no pain 2 h post-dose in 59.3% of patients in the AAC group versus 32.8% of patients in the placebo group.
Goldstein et al., 2006 [44]	double-blind, randomized, placebo-controlled	1555 (AAC—669, IB—666, placebo—220)	Acetaminophen 250 mg + aspirin 250 mg + caffeine 65 mg (AAC) versus ibuprofen 200 mg (IB) versus placebo	Both active treatments were significantly better at relieving pain and associated migraine symptoms than placebo. AAC was superior to IB in terms of pain relief, pain intensity reduction and pain response to the drug. The median time to pain relief (all aspects) was 20 min shorter for AAC than for IB.
Goldstein et al., 2005 [45]	randomized, placebo- controlled	171 (AAC—69, sumatriptan—67, placebo—35)	acetaminophen 500 mg + aspirin 500 mg + caffeine 130 mg (AAC) versus sumatriptan 50 mg versus placebo	AAC was found to be more effective than sumatriptan in the early treatment of a migraine attack. This is indicated by a greater difference in the cumulative assessment of pain before and after drug administration. The cumulative assessment of pain included pain intensity, pain relief, sustained analgesic response, symptom relief, reliever medication use, disability relief and global efficacy rating.
Pini et al., 2012 [46]	double-blind, randomized	92 (264 migraine attacks were evaluated, 131 PCF and 133 sumatriptan)	paracetamol 1000 mg + caffeine 130 mg (PCF) versus sumatriptan 50 mg	Similar treatment effects (pain relief) and drug tolerance in the patients’ assessment.
Láinez et al., 2007 [47]	double-blind, crossover clinical trial	229 (EC—115, almotriptan—114)	ergotamine 2 mg + caffeine 200 mg (EC) versus almotriptan 12.5 mg	Almotriptan was more effective and safer than EC.
Peroutka et al., 2004 [48]	randomized, double-blind, placebo-controlled	72 (12 in each of 6 treatment sequences)	diclofenac sodium 100 mg with or without caffeine 100 mg versus placebo	Diclofenac in combination with caffeine was most effective in treating a migraine attack (in 41% of patients).
Baratloo et al., 2001 [49]	prospective quasi-experimental study	70 (35 patients in each group)	intravenous caffeine citrate 60 mg versus intravenous magnesium sulfate 2 g	Both medications significantly reduced the severity of migraine pain, but magnesium sulfate was more effective.

## Data Availability

Not applicable; the article is a review of previously published research.
